# Utilizing productivity and health breeding-to-market information along with disease diagnostic data to identify pig mortality risk factors in a U.S. swine production system

**DOI:** 10.3389/fvets.2023.1301392

**Published:** 2024-01-11

**Authors:** Edison S. Magalhães, Jeff J. Zimmerman, Pete Thomas, Cesar A. A. Moura, Giovani Trevisan, Kent J. Schwartz, Eric Burrough, Derald J. Holtkamp, Chong Wang, Christopher J. Rademacher, Gustavo S. Silva, Daniel C. L. Linhares

**Affiliations:** ^1^Department of Veterinary Diagnostic and Production Animal Medicine, College of Veterinary Medicine, Iowa State University, Ames, IA, United States; ^2^Iowa Select Farms, Iowa Falls, IA, United States; ^3^Department of Statistics, College of Liberal Arts and Sciences, Iowa State University, Ames, IA, United States

**Keywords:** swine, wean-to-finish, diagnostic data, risk factors, mortality

## Abstract

Aggregated diagnostic data collected over time from swine production systems is an important data source to investigate swine productivity and health, especially when combined with records concerning the pre-weaning and post-weaning phases of production. The combination of multiple data streams collected over the lifetime of the pigs is the essence of the whole-herd epidemiological investigation. This approach is particularly valuable for investigating the multifaceted and ever-changing factors contributing to wean-to-finish (W2F) swine mortality. The objective of this study was to use a retrospective dataset (“master table”) containing information on 1,742 groups of pigs marketed over time to identify the major risk factors associated with W2F mortality. The master table was built by combining historical breed-to-market performance and health data with disease diagnostic records (Dx Codes) from marketed groups of growing pigs. After building the master table, univariate analyses were conducted to screen for risk factors to be included in the initial multivariable model. After a stepwise backward model selection approach, 5 variables and 2 interactions remained in the final model. Notably, the diagnosis variable significantly associated with W2F mortality was porcine reproductive and respiratory syndrome virus (PRRSV). Closeouts with clinical signs suggestive of *Salmonella* spp. or *Escherichia coli* infection were also associated with higher W2F mortality. Source sow farm factors that remained significantly associated with W2F mortality were the sow farm PRRS status, average weaning age, and the average pre-weaning mortality. After testing for the possible interactions in the final model, two interactions were significantly associated with wean-to-finish pig mortality: (1) sow farm PRRS status and a laboratory diagnosis of PRRSV and (2) average weaning age and a laboratory diagnosis of PRRS. Closeouts originating from PRRS epidemic or PRRS negative sow farms, when diagnosed with PRRS in the growing phase, had the highest W2F mortality rates. Likewise, PRRS diagnosis in the growing phase was an important factor in mortality, regardless of the average weaning age of the closeouts. Overall, this study demonstrated the utility of a whole-herd approach when analyzing diagnostic information along with breeding-to-market productivity and health information, to measure the major risk factors associated with W2F mortality in specified time frames and pig populations.

## 1 Introduction

The increasing implementation of information technologies in the swine industry has resulted in an abundance of diverse, but usually disconnected, data streams recording most of the aspects of raising pigs, e.g., productivity, health, environment, diagnostic, logistics, and infrastructure. If the data streams are connected, producers have the potential to measure the effect of specific risk factors on performance under the specific production system’s conditions and, thereafter, tailor swine health and production management to specific conditions and swine populations/pig flows.

Swine wean-to-finish (W2F) mortality is a key performance indicator (KPI) in modern swine production, and it is the result of interactions among multiple infectious and non-infectious agents (1, 2) which involve the epidemiological triad of disease, i.e., a dynamic interaction between pathogen, host, and environmental characteristics occurring sequentially from birth-to-market (~6 months) that are constantly changing over time. Although most swine producers collect information relevant to these factors, the data is usually being stored in multiple formats and scattered across different software or files (3).

An example of a data stream available to many swine production systems is diagnostic information from veterinary diagnostic laboratories (VDL’s). Veterinarians and producers collect and submit samples to VDL’s to diagnose cause(s) of disease, to monitor health, and/or respond to immediate disease threats. Diagnostic result is generally utilized immediately after the final diagnostic by swine veterinarians for utility in intervention decisions, and then stored in data files without further application. In fact, the aggregate retrospective diagnostic results collected over the lifetime of multiple closeouts (cohorts) represents a potentially important data source as demonstrated in other studies (4–7).

In this study, productivity, health, and diagnostic laboratory data were integrated to analyze the association between growing pig performance and wean-to-finish mortality (W2F). Thus, the objective of this study was to characterize associations between disease and mortality in groups of growing pigs, along with relevant health and performance observations from birth-to-market.

## 2 Materials and methods

### 2.1 Overview

A retrospective study was conducted on wean-to-finish (W2F) mortality for 1,742 “closeouts.” A “closeout” was defined as a group of pigs marketed within one United States commercial swine production system between January 2018 and June 2019. A closeout may have originated from either wean-to-finish sites or nursery followed by finisher sites. The outcome variable of interest was the number of pigs in each closeout that died in the growing phase as a proportion of those placed on feed. Closeout data available for the analysis are described in Table 1 and included 18 variables concerning the breed-to-market phase of production, i.e., disease status (e.g., PRRSV) and production performance data from the breeding herd(s) of origin, placement history (single-stocked or double-stocked groups; wean-to-finish flow or nursery followed by finisher), post-weaning productivity & health, and diagnostic data for each diagnostic case that included tissues and originated from the closeouts in this study. The columns of each variable included on Table 1 represents the categories within each variable.

**Table 1:** Data dictionary for variables included in the analyses.

Variable name	Category A	Category B	Category C	Category D
Dx Code – assigned^1^	>1 Dx Code assigned	No Dx Code assigned	–	–
Dx Code – diversity^2^	Single etiology	Multiple etiologies	No Dx Code	–
Dx Code – frequency^3^	Single submission	Multiple submission	No Dx Code	-
Dx Code - age of submission^a^	Early nursery	Late nursery	Finisher	No Dx Code
PRRSV Dx Code	Diagnosed	Other diagnosis*	No Dx Code†	-
Influenza Dx Code	Diagnosed	Other diagnosis*	No Dx Code†	-
*S. suis* Dx Code	Diagnosed	Other diagnosis*	No Dx Code†	-
*P. multocida* Dx Code	Diagnosed	Other diagnosis*	No Dx Code†	-
*E. coli* Dx Code	Diagnosed	Other diagnosis*	No Dx Code†	-
*Salmonella* spp. Dx Code	Diagnosed	Other diagnosis*	No Dx Code†	-
Rotavirus Dx Code	Diagnosed	Other diagnosis*	No Dx Code†	-
*G. parasuis* Dx Code	Diagnosed	Other diagnosis*	No Dx Code†	-
Sow farm PRRSV status^d^	Negative	Endemic	Epidemic	-
Avg. weaning age^b^	Q1 (x̄ = 15.3 days)	Q2 (x̄ = 16.8 days)	Q3 (x̄ = 17.9 days)	Q4 (x̄ = 20.3 days)
Avg. pre-weaning mortality^b^	Q1 (x̄ = 10.7%)	Q2 (x̄ = 13.1%)	Q3 (x̄ = 14.8%)	Q4 (x̄ = 18.0%)
Enteric disease^c^	*E. coli* and *Salmonella*	*E. coli*	*Salmonella*	No report
Production type	Double-stock	Single-stock	–	–
Pig flow	Wean-to-finish	Nursery → Finisher	–	–

^1^Closeouts with or without Dx Code information available; ^2^number of pathogens diagnosed from Dx Code data during the closeouts’ life cycle; ^3^number of submission of samples to the Veterinary Diagnostic Laboratory during the closeouts’ life cycle.

^a^Dx Code age: early nursery (first 1–3 weeks post-weaning); late nursery (3–7 weeks post-weaning); finisher (>7 weeks). ^b^“Q” refers to quartile and the quartile mean (x̄) for the variable. ^c^“Enteric disease” based on report(s) from field veterinarians. ^d^Wild-type PRRSV status of sow farms originating the closeouts, based on ongoing herd monitoring. PRRSV “Epidemic” = herds within the first 16 weeks after a PRRSV outbreak; “Endemic” = week 17 after an outbreak until negative results of wt-PRRS; “Negative” = farms with negative detection of wild type PRRSV but vaccinated for PRRSV using a PRRSV MLV vaccine. ^*^Other diagnosis = other pathogen(s) diagnosed but not the etiology described. ^†^No Dx Code = closeouts without submission of tissue for diagnosis.

### 2.2 Diagnostic data

Diagnostic evaluations were performed at the Iowa State University Veterinary Diagnostic Laboratory (ISU VDL, Ames, Iowa). Diagnostic data from closeouts’ cases with samples submitted for diagnosis at the ISU VDL included clinical information, laboratory assays performed, evaluation of macroscopic and/or microscopic lesions, and the diagnostic code (Dx Code) ultimately assigned to each tissue case by the veterinary diagnostician. Diagnostic data from non-tissue submissions were not included in this study.

The ISU VDL disease diagnostic coding system (Dx Code) is used to succinctly summarize pathologic process(s) and etiology(s) found in submissions of tissues from diseased pigs, used to describe diagnostic alignment between pathogens detected and lesions observed in tissues submitted for diagnostic evaluation. These codes are organized in terms of body system affected/insult type/predominant lesions(s) detected/confirmed or highly likely specific agent(s), and provide a precise etiological diagnosis when data aligns (8). One or more DX codes are assigned per case depending on these evaluations. For example, a case containing a lung tissue submitted for evaluation with a clinical background of respiratory disease, macroscopic and/or microscopic lesions compatible with *Mycoplasma hyopneumoniae* (*Mhp*) infection, and a PCR positive result for *Mhp* would have a Dx Code of respiratory/bacterial/ pneumonia/*Mycoplasma hyopneumoniae*. Notably, despite the tests utilized for each case being expected to be different, the final Dx Code is provided in a standardized format for all cases. All Dx Codes assigned to a closeout were included in the final master table, as well as closeouts that had two or more Dx Codes from multiple case submissions over the growing period or from one submission with multiple pathogens, as described in Table 1 as Dx Code frequency and Dx Code diversity, respectively.

### 2.3 Sow farm PRRSV status and productivity variables

Breeding herds were classified as PRRSV epidemic for the first 16 weeks after a PRRSV outbreak and then as “endemic” from week 17 until they were classified as “negative” based on the absence of wild-type PRRSV RNA detection and absence of clinical signs consistent with PRRSV infections. Notably, PRRSV negative farms are not considered naïve as the presence of PRRSV antibodies in the sows is expected. The major difference compared to the epidemic and endemic status is the absence of clinical signs combined with negative results for PRRSV. All herds were vaccinated with commercial modified live virus (MLV) PRRSV vaccine and detection of vaccine-like PRRSV did not disqualify negative herds. Average pre-weaning mortality and average weaning age were the remaining variables related to the breeding herd included in this study and described the average performance of the cohort of weaned pigs moved to the growing phase. When multiple weaned groups (i.e., from multiple sow farms) are placed together in growing sites, the weighted average was calculated for the variables mentioned above based on each farm performance and the number of animals from each origin. These variables were categorized by quartiles and the mean of the variables in the continuous format was reported in Table 1.

### 2.4 Data characteristics and management

SAS® Version 9.4 (SAS Institute, Inc., Cary, NC) was utilized to build algorithms used to import, manage, and integrate the production data and diagnostic data for 1,742 closeouts from a commercial swine production system, as fully described by (9). Data cleaning was performed after aggregating the data streams into the master table, i.e., closeouts with missing data were excluded from the final dataset. This process produced a single master table containing 1,720 closeouts (~5,000,000 pigs) marketed from January 2018 to June 2019, which was utilized on the analyzes of the multivariable model.

### 2.5 Statistical analyses

The final master table contained retrospective data from 1,720 closeouts and 18 variables representing diagnostic data, sow farm factors, and growing phase characteristics (Tables 1, 2). Initially, to meet the assumption of normality the outcome was log-transformed, and the assumption was tested using the Shapiro–Wilk test. Thereafter, the 18 variables were evaluated separately for their association with W2F mortality using a univariate linear mixed model (SAS PROC GLIMMIX) with the sow farm(s) from which the pigs in the closeout originated as a random effect. While informative, univariate analyses have well-recognized limitations, e.g., confounding, that can be addressed using multivariable models.

**Table 2:** Variables captured in the master-table and their values in each data analysis step.

Data stream	Variable	Univariate analysis	Initial multivariable analysis	Final multivariable analysis^c^
Dx Code	Dx Code - assigned	<0.0001	NI^a^	NI
	Dx Code - diversity	<0.0001	0.2206	NS^b^
	Dx Code - frequency	<0.0001	0.4320	NS
	Dx Code - age of submission^a^	<0.0001	0.7662	NS
	PRRSV Dx Code	<0.0001	<0.0001	<0.0001
	Influenza A virus Dx Code	<0.0001	0.6585	NS
	*Strep. suis* Dx Code	<0.0001	0.1458	NS
	*P. multocida* Dx Code	<0.0001	0.7399	NS
	*E. coli* Dx Code	<0.0001	0.3861	NS
	*Salmonella* Dx Code	<0.0001	0.6544	NS
	Rotavirus Dx Code	<0.0001	0.5988	NS
	*G. parasuis* Dx Code	<0.0001	0.7135	NS
Sow Farm	Avg. weaning age	0.0003	<0.0001	0.0027
	Avg. pre-weaning mortality	<0.0001	<0.0001	<0.0001
	Sow farm PRRSV status	<0.0001	<0.0001	<0 0.0001
Growing phase	Enteric challenge	<0.0001	0.0218	0.0042
	Production type	0.8497	NS	NS
	Pig flow	0.0127	0.0725	NS

^a^NI – Variable not included in the initial multivariable analysis due to multicollinearity (VIF > 5). ^b^NS – Variables not selected for the multivariable analysis due to large value of *p* (*p* > 0.05). ^c^Interactions included in the model but not described in the table were: PRRSV status*PRRSV Dx Code (*p* = 0.0224); Weaning age*PRRSV Dx Code (*p* < 0.0001).

The initial multivariable model included all variables with a moderate association to W2F mortality (*p* < 0.10, univariate analysis). To achieve the final multivariable model, manual stepwise backward model selection was used to identify variables significantly associated with W2F mortality (*p* < 0.05) as demonstrated in Table 2. Thereafter, multicollinearity in the final model was assessed, and variables with a variance inflation factor (VIF) value >5 were excluded. Once the final multivariable model was built, Tukey–Kramer pairwise comparisons were done to identify significant differences between levels of categorical variables.

## 3 Results

The geometric mean log-W2F mortality for the 1,720 closeouts in the study was 8.55%, (95% CI 8.37, 8.73%). A total of 392 closeouts had >1 Dx Code(s), while 1,328 groups had no tissue submissions and, therefore, no Dx Codes. The results of the univariate analyses on Dx Code variables are presented in Table 3. “No Dx Code,” i.e., the category representing closeouts with no tissue submissions to the diagnostic laboratory, served as the baseline of comparison and denoted groups of pigs with absence of clinical signs to trigger diagnostic investigation.

**Table 3:** Results of the univariate analyses for Dx Code variables on wean-to-finish mortality.

Explanatory variables	Category (no. groups)^*^	Mortality	95% CI
Dx Code - assigned (*p* < 0.0001)	No Dx Code (1,328)	7.9%^b^	7.5%, 8.4%
	>1 Dx Code(s) (392)	10.1%^a^	9.5%, 10.8%
Dx Code - frequency (*p* < 0.0001)	Single submission (365)	10.0%^b^	9.4%, 10.7%
	≥2 submissions (69)	11.1% ^a^	9.9%, 12.4%
	No Dx Code (1,328)	7.9%^b^	7.5%, 8.4%
Dx Code - diversity (*p* < 0.0001)	Single etiology (226)	9.8%^a^	9.1%, 10.5%
	Multiple etiologies (208)	10.6%^a^	9.8%, 11.4%
	No Dx Code (1,328)	7.9%^b^	7.5%, 8.4%
Dx Code submission age (*p* < 0.0001)	Early nursery (102)	10.6%^a^	9.6%, 11.6%
	Late nursery (258)	10.2%^b^	9.5%, 10.9%
	Finisher (74)	9.4%^bc^	8.4%, 10.5%
	No Dx Code (1,328)	7.9%^b^	7.5%, 8.4%
PRRS Dx Code (*p* < 0.0001)	Diagnosed (160)	9.3%^a^	8.7%, 9.9%
	Other diagnosis (232)	8.2%^b^	7.7%, 8.7%
	No Dx Code (1,328)	7.9%^c^	7.5%, 8.4%
IAV Dx Code (*p* < 0.0001)	Diagnosed (81)	9.7%^a^	8.8%, 10.7%
	Other diagnosis (311)	10.3%^a^	9.6%, 10.9%
	No Dx Code (1,328)	7.9%^b^	7.5%, 8.4%
*Strep. suis* Dx Code (*p* < 0.0001)	Diagnosed (45)	11.4%^a^	10.1%, 12.8%
	Other diagnosis (347)	10.0%^a^	9.4%, 10.7%
	No Dx Code (1,328)	7.9%^b^	7.5%, 8.4%
*P. multocida* Dx Code (*p* < 0.0001)	Diagnosed (23)	11.1%^a^	9.5%, 13.1%
	Other diagnosis (369)	10.1%^a^	9.5%, 10.8%
	No Dx Code (1,328)	7.9%^b^	7.5%, 8.4%
*G. parasuis* Dx Code (*p* < 0.0001)	Diagnosed (58)	10.8%^a^	9.7%, 12.1%
	Other diagnosis (334)	10.0%^a^	9.4%, 10.7%
	No Dx Code (1,328)	7.9%^b^	7.5%, 8.4%
*E. coli* Dx Code (*p* < 0.0001)	Diagnosed (47)	9.4%^a^	8.3%, 10.6%
	Other diagnosis (345)	10.2%^a^	9.6%, 10.9%
	No Dx Code (1,328)	7.9%^b^	7.5%, 8.4%
*Salmonella* Dx Code (*p* < 0.0001)	Diagnosed (24)	10.5%^a^	8.9%, 12.3%
	Other diagnosis (368)	10.1%^a^	9.5%, 10.8%
	No Dx Code (1,328)	7.9%^b^	7.5%, 8.4%
Rotavirus Dx Code (*p* < 0.0001)	Diagnosed (60)	10.1%^a^	9.1%, 11.3%
	Other diagnosis (332)	10.2%^a^	9.5%, 10.8%
	No Dx Code (1,328)	7.9%^b^	7.5%, 8.4%

^abc^Different superscript letters indicate significant differences through pairwise comparisons (Tukey–Kramer test, *p* < 0.05) by comparing the least square means estimates of the back-transformed log-W2F mortality.

^*^“No Dx Code” category represents closeouts with no tissue submissions to the veterinary diagnostic. Laboratory, serving then as the baseline for comparison.

In summary, the univariate analysis revealed a notable disparity in W2F mortality (10.1% vs. 7.9%) between closeouts with more than one Dx Code and those without any Dx Code (refer to Table 3). When considering submission age, the average W2F closeout mortality rates were 10.6, 10.2, and 9.4%, respectively, for cohorts with Dx Codes in early nursery, late nursery, and finisher stages. Additionally, in the comparison between closeouts with a single submission and those with more than two submissions, the mean W2F mortality rates were 10.0 and 11.1%, respectively. Similarly, when evaluating “single pathogen” versus “multiple pathogens,” the mean W2F mortality rates were 9.8 and 10.6%, respectively.

From the univariate analyses, 17 of 18 variables were included in the initial multivariable model. Manual stepwise model selection resulted in the exclusion of 12 variables that did not meet the eligibility criteria (*p* < 0.05) and one variable due to multicollinearity (VIF > 5), as demonstrated in Table 2. The final multivariable model included PRRS Dx Code, sow farm PRRSV status, average weaning age, average pre-weaning mortality, and enteric disease (Table 4), and the final VIF values are available in Supplementary Table S1.

**Table 4:** Results of the final multivariable analysis on wean-to-finish mortality.

Explanatory variables (no. groups)^†^	Mortality^*^	95% CI	*p* value
PRRS Dx Code (1,720)			<0.0001
Dx Code - PRRS (160)	13.5%^a^	12.1–15.1%	
Dx Code - Other (not PRRS) (232)	10.3%^b^	9.4–11.4%	
Dx Code - None (1,328)	9.0%^c^	8.2–9.9%	
Sow farm PRRSV status (1,720)			<0.0001
Epidemic (484)	11.8%^a^	10.6–13.2%	
Endemic (859)	10.5%^b^	9.2–11.2%	
Negative (377)	10.1%^b^	9.4–11.7%	
Avg. pre-weaning mortality (1,720)			<0.0001
Q1–10.7% (404)	10.4%^a^	9.4–11.5%	
Q2–13.1% (440)	10.2%^a^	9.3–11.3%	
Q3–14.8% (443)	10.6%^a^	9.6–11.7%	
Q4–18.0% (433)	12.0%^b^	10.9–13.3%	
Avg. weaning age (1,720)			0.0032
Q1–15.3 days (423)	10.9%^ab^	9.9–12.2%	
Q2–16.8 days (432)	11.7%^a^	10.5–13.0%	
Q3–17.9 days (428)	10.4%^b^	9.4–11.6%	
Q4–20.3 days (437)	10.1%^b^	9.1–11.3%	
Enteric disease (1,720)			0.0050
*E. coli* and *Salmonella* (12)	12.7%^a^	10.1–15.7%	
*E. coli* (27)	11.1%^ab^	9.6–12.8%	
*Salmonella* (21)	10.4%^ab^	8.7–12.4%	
No report (1660)	9.4%^b^	8.8–10.0%	

^*^Geometric mean log-transformed W2F mortality estimates derived from the multivariable model represents the average value for the group of closeouts marketed in each variable category.

^abc^Different superscript letters indicate significant differences (Tukey test, *p* < 0.05). ^†^Interactions included in the model but not described in the table were: Sow farm PRRSV status*PRRSV Dx Code (*p* = 0.0224) and Avg. weaning age*PRRSV Dx Code (*p* < 0.0001). These interactions are better described in Figures 1, 2.

Q1-Q4 represents the mean value for each quartile.

As shown in Table 4, groups with PRRS Dx Codes, groups diagnosed with other pathogens (not PRRS), and groups with No Dx Code had W2F mortalities of 13.5, 10.3 and 9.0%, respectively. The final multivariable analysis demonstrated that “Dx Code - PRRS” was the only significant diagnosis, i.e., the pathogens identified as statistically significant in the univariate model were confounded by PRRSV. To verify this, interactions between Dx Code - PRRS and each of the other pathogen-specific Dx Codes were tested separately in the final multivariable model, but none were significant. Also, the W2F mortality values for PRRS Dx Code categories changed when comparing the univariate and the multivariable results, which is a consequence of adjusting the estimates according to the remaining covariates included in the final multivariable model.

Sow farm PRRSV status at the time the cohorts were weaned was identified as a significant factor through the model selection process. Weaned groups originating from sow farms with epidemic PRRSV status subsequently had higher downstream W2F mortality (11.8%) versus cohorts from PRRSV endemic (10.5%) or negative sow farms (10.1%).

Average pre-weaning mortality on the sow farm was also significant, i.e., the observed W2F mortality for groups weaned with pre-weaning mortality quartiles of 10.7, 13.1, 14.8, and 18.0%, were 10.4, 10.2, 10.6, and 12.0%, respectively. In other words, closeouts that originated from sow farms where the average pre-weaning mortality was high were associated with higher downstream W2F mortality as well. Weaning age was likewise statistically associated with the downstream W2F mortality. As weaning age quartiles increased (15.3, 16.8, 17.9, and 20.3 days of age at weaning), W2F mortality decreased (10.9, 11.7, 10.4, and 10.1%).

A growing phase variable that remained significant in the multivariable model was the determination of enteric disease in the growing phase by the herd veterinarian. Specifically, the mean W2F mortality for groups without clinically apparent enteric disease was 9.4%, while mortality in groups with clinical disease typical of *E. coli*, *Salmonella*, or both *E. coli* and S*almonella* was 11.1, 10.4, and 12.7%, respectively.

**Figure 1:**
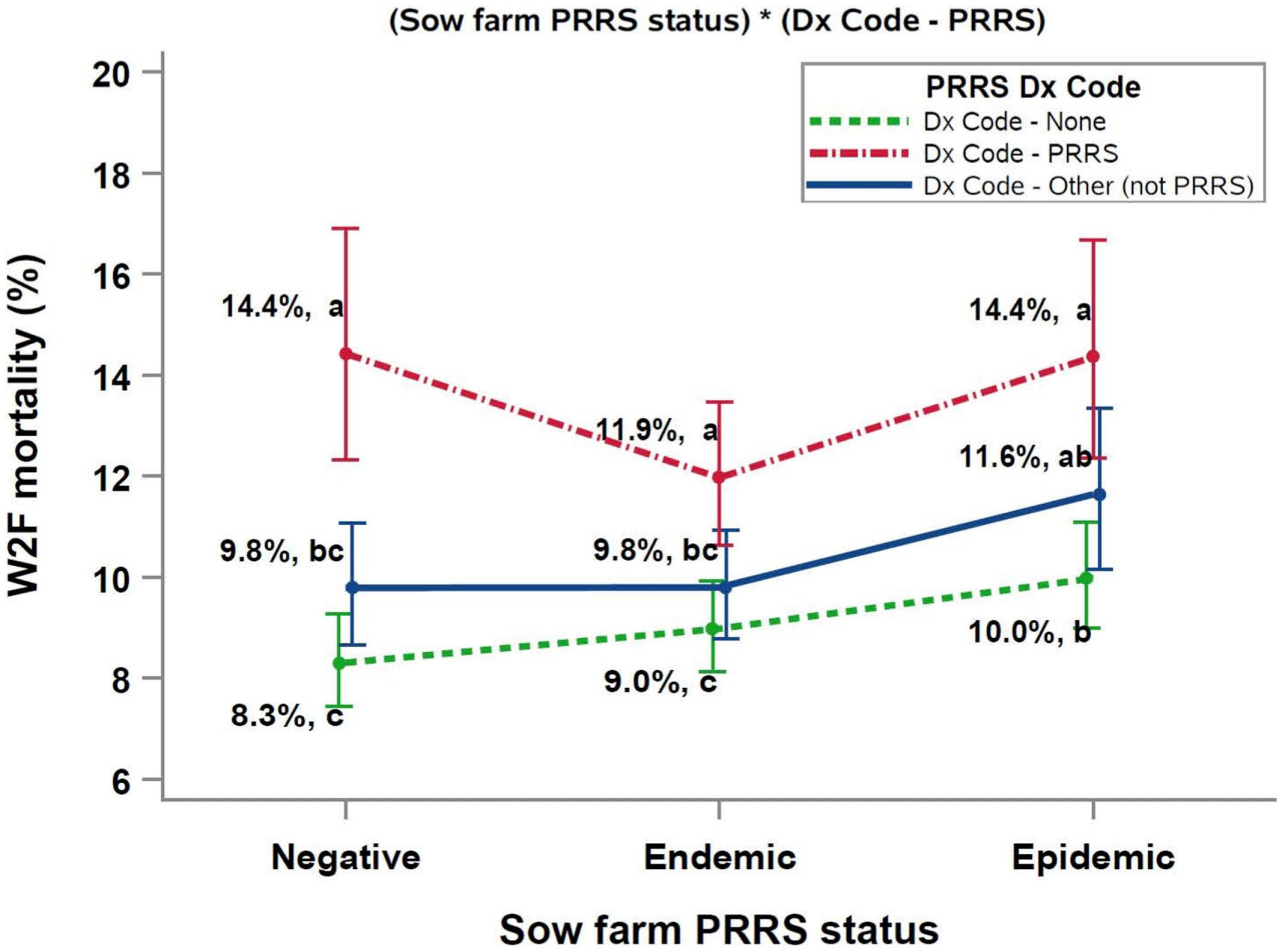
Interaction between Sow farm PRRSV status and PRRSV diagnosis Dx Code data. ^abc^Different superscript letters indicate significant differences (Tukey test, *p* < 0.05). PRRS, porcine reproductive and respiratory syndrome; W2F, wean-to-finish.

**Figure 2:**
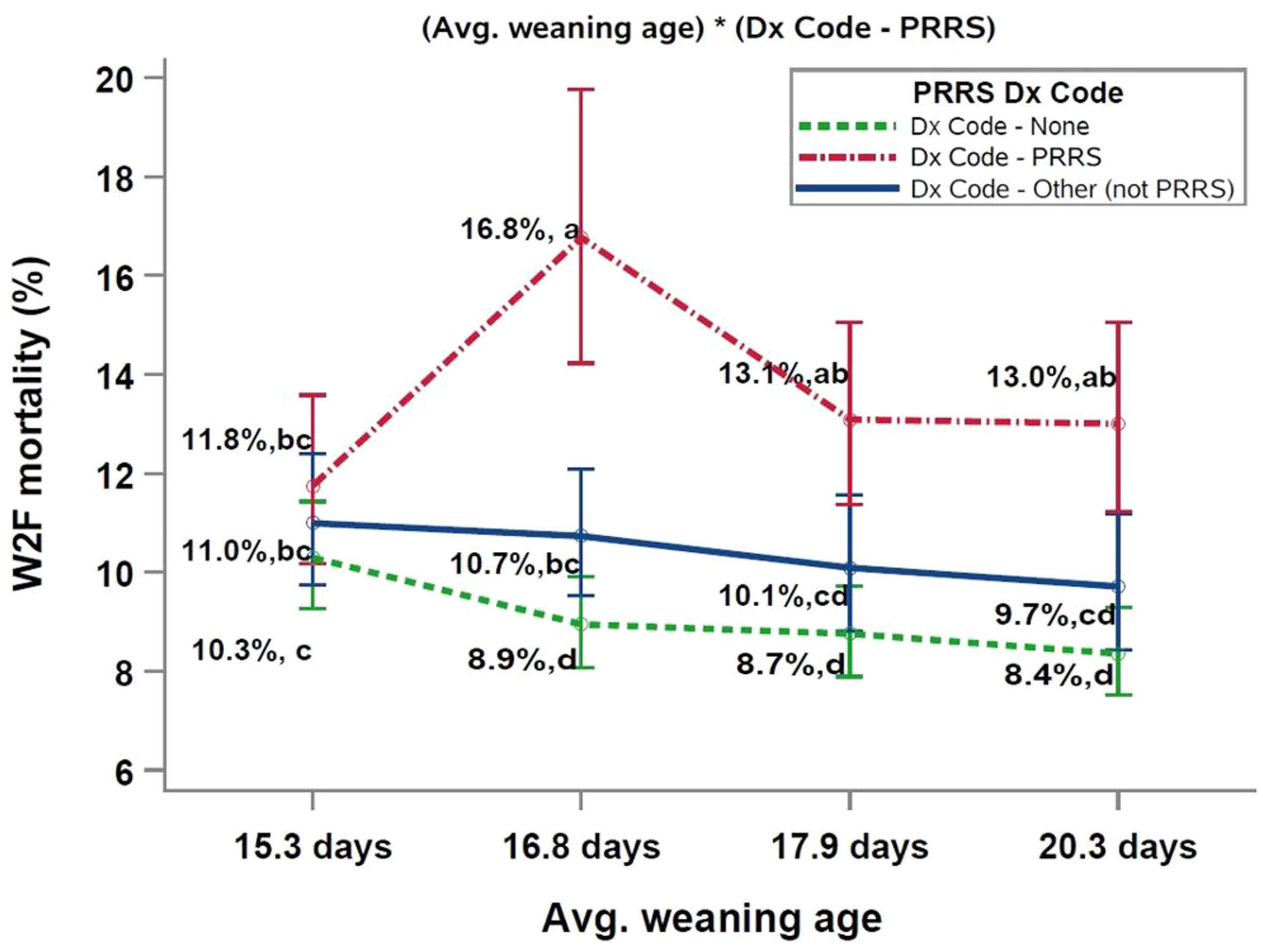
Interaction between PRRSV Dx Code data and average weaning age of the closeouts. ^abc^Different superscript letters indicate significant differences (Tukey test, *p* < 0.05). PRRS, porcine reproductive and respiratory syndrome; W2F, wean-to-finish.

The two significant interactions included in the final multivariable model were (PRRS Dx Code*sow farm PRRSV status), and (Dx Code - PRRSV*average weaning age), as shown in Figures 1, 2, respectively.

The relationship between sow farm PRRSV status, PRRS Dx Code, and W2F mortality was complex (Figure 1). Overall, the diagnosis of PRRSV during the growing period resulted in both numeric and statistically significant increases in mortality compared to groups with “Dx Code - None” or “Dx Code - Other (not PRRS).” The highest mortalities were observed in closeouts with a PRRS diagnosis (Dx Code – PRRS) weaned from PRRSV epidemic and negative sow farms. Further, diagnosing a pathogen other than PRRSV resulted in a numeric (but not a significant) increase in mortality compared to closeouts with no Dx Codes across all sow farm PRRS status categories.

The analysis of W2F mortality by weaning age showed a similar trend (Figure 2). That is, closeouts classified as “Dx Code – None” had the lowest mortality followed closely by closeouts classified as “Dx Code other - (not PRRS).” Thus, the highest W2F mortality was observed in “Dx Code – PRRS” closeouts. Declining mortality with increased weaning age was observed for the “Dx Code – None” and “Dx Code - other (not PRRS)” categories. For both categories, the lowest W2F mortality was observed in groups weaned in the oldest weaning age quartile (20.3 days). On the other hand, the highest mortality occurred in the youngest weaning age quartile (15.3 days). Thus, in the absence of a PRRS diagnosis in the growing phase, weaning age was an important risk factor for W2F mortality. Conversely, PRRS diagnosis in the growing phase negates the weaning age effect and results in higher mortality across all weaning ages compared to groups without PRRS diagnosis.

## 4 Discussion

Writing at the time when the swine industry began to transition from many herds with relatively few animals on each premise to fewer farms with larger populations, (10) described the need to move from single-agent causality to multifactorial causes. Today, identifying and addressing the multiple factors that drive pig mortality is the basis of effective disease control and prevention, as well as a key to the economic viability of swine enterprises. To achieve this requires taking a holistic or whole-herd approach when investigating swine health issues. For the wean-to-finish phase of production, this approach is increasingly feasible because of the availability of performance and health data collected routinely over the course of the pigs’ production cycle (pre and post-weaning phase), as has been previously described in other observational studies (9, 11–18). In this study, the whole-herd approach was achievable because the data streams included premise identification and dates for each recorded event. These unique identifiers and the use of SAS algorithms allowed automated data wrangling and the development of a master table containing retrospective information for each closeout marketed in the study period. This study also demonstrated the power of capturing and merging multiple birth-to-market data streams (whole-herd approach) in terms of revealing the impact of “distant” spatiotemporal events, e.g., sow herd health status, on downstream grower pigs. That is, combining diagnostic data with other data streams made it possible to more fully understand health dynamics by connecting earlier events to current disease problems.

Diagnostic data is generally utilized to estimate health status and disease pressure at a point in time, thus guiding producers and veterinarians as they respond to protect the health and productivity of the population (8). This was confirmed in the present study as W2F mortality was higher for groups with diagnostic codes assigned compared to flows without diagnostic codes, indicating that the submission of tissues for diagnosis was associated with evidence of disease expression at the population level, therefore being a proxy of disease activity in growing pigs.

A diagnosis is an assessment based on the pertinent cumulative information, i.e., herd history, geographical location, clinical signs, lesions, and test results, available for each case. Overall, this study demonstrated that the standalone interpretation of Dx Code results, e.g., univariate analyses or pivot tables, should be done cautiously because such estimates are not adjusted for important confounders (19). Thus, various Dx Code explanatory variables were significant in the univariate analyses (Table 3), but a multivariable model that accounted for other covariates and confounders revealed that only one (PRRS Dx Code) was significant for W2F mortality. In particular, in the absence of PRRS, other pathogens were not significantly associated with W2F mortality but their importance for other W2F metrics, e.g., average daily weight gain (ADWG), cannot be ruled out, as demonstrated in previous studies on *Mhp* (20–22), PEDV (23) and influenza A virus (24, 25).

Among the five variables in the final multivariable model, three concerned productivity and health in the pre-weaning phase of production. This confirmed the importance of breeding herd characteristics on the downstream performance of growing pigs, as previously shown in other studies (26–31). Consistent with the literature, the multivariable model indicated that the impact of sow farm factors on growing pig mortality is complex, with PRRSV infection meriting special consideration both on the sow farm and subsequently in growing pigs (32–40). For example, closeouts originating from PRRS epidemic sow farms, i.e., cohorts weaned within the first 16 weeks after a Sow farm PRRSV outbreak, had the highest downstream mortality rates.

The remaining two variables in the multivariable model represented PRRS infection (PRRS Dx Code) and enteric challenge in the post-weaning phase of production. Closeouts diagnosed with PRRS in the growing phase, i.e., Dx Code – PRRS, had significantly higher W2F mortality compared to closeouts classified as “Dx Code – Other (not PRRS)” or no diagnosis (“Dx Code – None”). Similarly, a previous growing pig study in “pig-dense” areas reported that >90% of the groups detected wild-type PRRSV-2 in ≥1 sampling (37), indicating high virus circulation in growing pigs. Furthermore, groups with reported enteric challenges suggestive of both *E. coli* and *Salmonella* had higher W2F mortality than those without enteric challenges. Unfortunately, the precise cause of enteric disease was not always confirmed by diagnostic testing in this study, and thus, the specific contributions of either agent or other causes of enteric disease cannot be determined and warrant further investigation in future analyses. Other investigators have reported similar patterns for PRRS and enteric challenges on W2F mortality (16, 41–45).

An analysis of the interaction between closeouts diagnosed with PRRS in the growing phase (PRRS Dx Code) and the PRRSV status of the sow farm at the cohorts’ time of weaning (Figure 1) highlighted the importance of PRRSV as a risk factor. That is, cohorts with a PRRSV diagnosis had the highest mortality across all categories, particularly groups weaned from sow farms with either a PRRSV negative or PRRSV epidemic status. These results suggested that grower pigs from PRRSV wild-type negative sow farms may have had no prior exposure to wild-type PRRSV and were, therefore, less able to respond immunologically to a wild-type PRRSV infection in the growing phase, even though negative sow farms were all vaccinated for PRRSV, similarly to the PRRS stable Category 2-vx sow farms described by (46). On the other hand, the poor performance of pigs weaned from epidemic sow farms implies that they may have been suboptimal in terms of immunity as a consequence of the clinical manifestation of PRRS in sow farms through the acute phase after the outbreak (47). In contrast to PRRSV epidemic and wild-type negative sow farms, closeouts from PRRSV endemic sow farms had numerically lower mortality, presumably because these pigs had the advantage of maternal immunity against PRRSV, but this assumption can only be confirmed by analyzing potential confounders not included in this study such as the different PRRS virus strains and/or the presence of multiple strains in a farm. A key observation from this analysis was that, even in the absence of a PRRS Dx Code, both the “Dx Code - None” and “Dx Code – Other (not PRRS)” categories had higher W2F mortality when the closeouts originated from PRRSV epidemic sow farms.

Likewise, the interaction between PRRS Dx Code and the average weaning age of the groups (Figure 2) demonstrated similar results, i.e., groups with PRRS “Dx Code – PRRS” had higher W2F mortality values, independent of the weaning age categories. On the other hand, a trend toward numerically higher W2F mortalities was observed for groups with younger average weaning age and in the absence of PRRS diagnosis in the growing phase. For closeouts without any diagnosis throughout the growing phase (Dx Code – None), closeouts weaned from the younger weaning age category (15.3 days) had 1.4, 1.6, and 1.9% higher W2F mortality when compared to the remaining weaning age categories (16.8 days, 17.9 days, and 20.3 days, respectively). Others have also reported lower W2F mortality and better growth performance with increased average weaning age (16, 26, 28, 31, 48).

The limitations of this study primarily involve the diagnostic data. First, the tissue samples submitted to the veterinary diagnostic laboratory for evaluation were selected by individual veterinarians or field staff. Within the production system, it is reasonable to assume variability among veterinarians in the choice and timing of pigs/samples for submission. Furthermore, disease circulation in large populations is rarely homogeneous and also varies over time, thus limiting the external validity of this study to other populations of market pigs. Also, most of the tissues submitted for Dx Code in this study originated from point-in-time sampling collection(s), which lacks continuous diagnostic monitoring based on a standardized protocol for all processes involving the submission of tissues for diagnosis, thus, not being consistently applied across all closeouts. Regardless, integrating disease diagnosis information with other data streams, including the performance of the sow farms at the time growing groups were farrowed and weaned, can assist field veterinarians in identifying data-driven solutions intended to improve herd performance.

## 5 Conclusion

This study demonstrated the application of the whole-herd approach in identifying the major risk factors associated with W2F mortality based on the analysis of an integrated master table containing both disease diagnostic information and pre- and post-weaning data related to productivity and health. The differences between univariable and multivariable analyses illustrate that standalone data assessment, i.e., pivot tables or univariate analyses, should be avoided in favor of the whole-herd approach. Further, the multivariable analysis showed that PRRSV infection continues to impact pig health, productivity, and W2F mortality in commercial herds throughout the growing phase and that the sow farm plays a major role in the downstream survivability of growing pigs. Notably, the interactions revealed in the data analyzed in this study are expected to change over time or between production systems, thus, the process requires ongoing data integration and analysis.

## Data availability statement

The datasets presented in this article are not readily available because the data presented in this study are available on reasonable request from the corresponding author. The data are not publicly available due to privacy. Requests to access the datasets should be directed to edison@iastate.edu.

## Author contributions

EM: Conceptualization, Data curation, Formal analysis, Methodology, Writing – original draft, Writing – review & editing. JZ: Writing – original draft, Writing – review & editing. PT: Conceptualization, Writing – review & editing. CM: Conceptualization, Writing – review & editing. GT: Conceptualization, Writing – review & editing. KS: Conceptualization, Writing – review & editing. EB: Conceptualization, Writing – review & editing. DH: Investigation, Methodology, Writing – review & editing. CW: Data curation, Formal analysis, Validation, Writing – review & editing. CR: Investigation, Methodology, Writing – review & editing. GS: Data curation, Formal analysis, Investigation, Methodology, Validation, Writing – review & editing. DL: Conceptualization, Funding acquisition, Investigation, Methodology, Project administration, Supervision, Writing – review & editing.
