# Supplementary material for: Utilizing productivity and health breeding-to-market information along with disease diagnostic data to identify pig mortality risk factors in a U.S. swine production system
Source: Front Vet Sci. 2024 Jan 11;10:1301392. doi: 10.3389/fvets.2023.1301392 (PMC10808511; doi:10.3389/fvets.2023.1301392)
Supplement: Supplementary file 1 [file Table_1.DOCX]

Table A - VIF analysis of all predictors included in the final multivariable model

| **Label** | **Parameter Estimate** | **Standard Error** | **t Value** | **Pr > \|t\|** | **Tolerance** | **Variance Inflation** |
| --- | --- | --- | --- | --- | --- | --- |
|  |  |  |  |  |  |  |
| Sow farm PRRSV status | 0.01259 | 0.00175 | 7.21 | <.0001 | 0.89738 | 1.11435 |
| Enteric disease | -0.0107 | 0.00496 | -2.15 | 0.0317 | 0.99387 | 1.00617 |
| PRRS Dx Code | 0.01893 | 0.00184 | 10.3 | <.0001 | 0.99536 | 1.00466 |
| Avg. weaning age | -0.0009 | 0.00065 | -1.35 | 0.1774 | 0.97134 | 1.0295 |
| Avg. pre-weaning mortality | 0.00252 | 0.00047 | 5.39 | <.0001 | 0.89978 | 1.11138 |
